# Assessment and Management of Cognitive and Psychosocial Dysfunctions in Patients With Major Depressive Disorder: A Clinical Review

**DOI:** 10.3389/fpsyt.2018.00493

**Published:** 2018-10-11

**Authors:** Andrea Fiorillo, Bernardo Carpiniello, Serafino De Giorgi, Silvestro La Pia, Giuseppe Maina, Gaia Sampogna, Edoardo Spina, Alfonso Tortorella, Antonio Vita

**Affiliations:** ^1^Department of Psychiatry, University of Campania “Luigi Vanvitelli”, Naples, Italy; ^2^Department of Public Health, Clinical and Molecular Medicine, University of Cagliari, Cagliari, Italy; ^3^Department of Mental Health, ASL Lecce, Lecce, Italy; ^4^Department of Mental Health, ASL Napoli 3 Sud, Naples, Italy; ^5^AOU San Luigi Gonzaga, University of Turin, Turin, Italy; ^6^Department of Clinical and Experimental Pharmacology, University of Messina, Messina, Italy; ^7^Department of Psychiatry, University of Perugia, Perugia, Italy; ^8^Neuroscience Section, Department of Clinical and Experimental Sciences, University of Brescia, Brescia, Italy; ^9^Department of Mental Health, Spedali Civili Hospital, Brescia, Italy

**Keywords:** assessment tools, cognitive symptoms, full functional recovery, major depressive disorder, personal functioning

## Abstract

**Background:** Full functional recovery is defined as a state in which patients are again able to enjoy their usual activities, return to work, and take care of themselves, and it should represent the end goal of treatment in patients with major depressive disorder (MDD). Patients with MDD report many unmet needs, including residual cognitive symptoms, lack of improvement in psychosocial functioning and life satisfaction, even during mood symptom remission. In this paper, we aim to: (a) identify the available assessment tools for evaluating cognitive and psychosocial functioning in patients with MDD; (b) provide an overview of therapeutic options that can improve full functional recovery in MDD also by improving cognitive symptoms.

**Methods:** The relevant databases MEDLINE, ISI Web of Knowledge – Web of Science Index, Cochrane Reviews Library and PsychoINFO were searched for identifying papers on validated tools for the assessment of cognitive and personal functioning in patients with MDD.

**Results:** New assessment tools (such as the THINC-it TOOL, the COBRA, the SCIP-D, and the UPSA-D) have been developed for evaluating the cognitive dysfunction in MDD patients. Adopting these tools in the clinical routine practice is useful to evaluate the improvement in cognitive functioning and, therefore, the achievement of full functioning recovery. The optimal management of patients with MDD include the combination of pharmacological compounds and psychosocial interventions for achieving full functional recovery in patients with MDD.

**Conclusions:** Full functional recovery must be the target of any treatment programme for patients with MDD. In order to achieve this goal, it is necessary to develop personalized treatment and integrate psychosocial and psychopharmacological interventions.

## Introduction

Depression is a complex disorder with multiple symptomatological clusters, including emotional, cognitive, and physical symptoms (1). From 2005, a significant increase in the incidence of the disorder of almost 20% has been observed (2). In 2015, depressive disorders were the greatest contributor to non-fatal health loss (2, 3). The average lifetime prevalence of major depressive disorder (MDD) is estimated at 14.6% in high-income countries (4). Moreover, MDD represents the leading cause of disability burden worldwide (5), accounting for 2.5% of global Disability Adjusted Life Years lost (6), especially in women (2).

While in the past remission was considered the only clinical endpoint in the management of patients with MDD (7), more recently the concept of full functional recovery has been proposed as the ultimate therapeutic objective (8, 9). In fact, it is now clear that many patients with MDD who achieve symptomatic remission do not report a substantial improvement in psychosocial functioning and satisfaction with life (10, 11). Full functional recovery can be defined as a condition in which the patient starts to enjoy his/her usual activities again, returns to work and is able to take care of him/herself (12, 13). The achievement of full functional recovery in patients with MDD may be hampered by patient and illness-related factors. The former includes age, pre-morbid level of functioning, level of education, work condition, comorbidity with other psychiatric diseases, and other medical conditions. The illness-related factors include the severity of clinical episodes, the effectiveness of treatments, time to remission, maintenance and quality of remission (13–17).

The main unmet need in the treatment of patients with MDD, who have responded to classic antidepressants, is the presence of residual symptoms, such as lack of energy, concentration problems, and sleep disturbances (12). Cognitive symptoms (namely deficits in attention, memory, executive function, and processing speed) (18), which have been neglected for many years in the clinical management of mood disorders, may represent the link between symptomatic remission and functional recovery (19). Neurocognition is a core feature of depressive episodes; cognitive symptoms can limit patients' psychosocial functioning, and achieving “cognitive remission” has been claimed as a relevant goal in the treatment of MDD (20).

Although a good antidepressant therapy should not only aim to improve affective symptoms, but also cognitive symptoms, psychosocial functioning, work functioning, and quality of life (21), the majority of clinical studies on MDD evaluate the effectiveness of treatments on affective symptoms only (19). In fact, among the most frequently used tools to assess outcomes from MDD, only three of the top 20 explore functional domains, and these have been used in <5% of trials with patients with depression (14). Moreover, different tools are available for evaluating these dimensions, being different in structure, content, length, way of compilation and target population. In this manuscript, we aim to perform a clinical review on the recent assessment tools for evaluating cognitive and psychosocial functioning in patients with MDD. Finally, a critical insight on the translation from the evaluation to the appropriate treatment of cognitive symptoms is provided.

## Materials and methods

### Search strategy

The relevant databases MEDLINE, ISI Web of Knowledge—Web of Science Index, Cochrane Reviews Library and PsychoINFO were searched for papers published in the last 5 years. Previous years had already been covered by Bortolato et al. (22) (for assessment tools evaluating cognitive functioning in patients with MDD) and by Lam et al. (14) (for assessment tools evaluating psychosocial functioning in patients with MDD) and we aim to update their data with findings from more recent trials.

The key words “depressive disorder,” “major depressive disorder,” “depressed mood” matched with “cognitive symptoms,” “cognitive functioning,” “cognitive deficits,” “psychosocial functioning,” “work functioning,” “social functioning,” and “assessment tools” were entered in the relevant databases. Only papers written in English and published in peer-reviewed journals were included in our review.

The reference lists of all papers selected in the primary search were manually searched for other potential manuscripts. Recently published international guidelines on the management of patients with MDD were also searched. The results of the search were independently evaluated by two authors who have analyzed all relevant papers.

## Results

In the last years, new assessment tools have been developed for evaluating the cognitive dysfunction in MDD patients. In particular, in 2017 Harvey et al. (23) tested the psychometric validity of the “University of California San Diego Performance-based Skills assessment (UPSA)” in patients with MDD, bipolar disorder, mild cognitive impairment, Alzheimer's disease and healthy older adults. The UPSA has been originally developed to assess older, community-dwelling patients with schizophrenia or severe mental illness and it has been adapted to assess functional capacity in patients with MDD (23). Authors found that UPSA can provide clinically relevant information for the management of patients with MDD, since it measures the everyday functioning skills in different function domains. In fact, the UPSA composite score correlates with cognitive performance in the real-world of persons with MDD but it is not influenced from the clinical mood symptoms of depression (23).

In 2016, McIntyre et al. developed the THINC-it TOOL (24–26) which is available as an application for smartphones, tablets, and PC. It can be used to specifically assess the level of cognitive dysfunction in patients with depressive disorders. This tool requires ~10–15 min to be completed (25), and therefore can be easily implemented in clinical practice. Cognitive deficits measured by the THINC-it tool are associated with significant psychosocial impairment in MDD (27).

The Montreal Cognitive Assessment (MoCA) is a brief screening tool originally developed for assessing the most common neurocognitive deficits in patients with mild cognitive impairment (28). Recently, it has been tested in a sample of patients with MDD, showing a valid and reliable proprieties with good internal consistency.

When assessing cognitive symptoms, it is essential to differentiate between objective and subjective cognitive deficits (such as memory or concentration complaints), since the correct identification of objective dysfunctions is necessary for monitoring the effects of pharmacological and non-pharmacological treatments. To this end, two new assessment tools have been validated recently, the Screen for Cognitive Impairment in Psychiatry-Depression (SCIP-D) and the Cognitive Complaints in Bipolar Disorder Rating Assessment (COBRA) (29). In particular, the SCIP requires <20 min to be completed and assesses verbal learning, working memory, verbal fluency, delayed memory and processing speed; while COBRA evaluates the subjective dimensions of cognitive complaints. These two instruments, originally developed for patients with bipolar disorders, have shown good psychometric properties and can be easily administered to patients with MDD.

Among the instruments for the evaluation of social functioning in patients with MDD, extensively reported by Lam et al. (14), it has been recently developed the “Recovery Index” (RI) (30). This instrument is based on the combination of the WSAS and Q-LES-Q scales, and it provides information on social, personal, and work functioning (30). The index can be easily calculated by accessing a web platform and entering the mean scores obtained by the patient at the WSAS and at the Q-LES-Q. In particular, a higher score at the RI means a higher level of functional recovery. This index has good psychometric properties, it is easy to use, and can be adopted in clinical and research settings. The details of all instruments are reported in Table 1.

**Table 1:** Assessment tools for evaluating cognitive and global functioning in patients with MDD.

**References**	**Acronym**	**Assessment tool**	**Characteristics**	**Target**	**Time need to be completed**
Srisurapanont et al. (28)	MoCA	Montreal cognitive assessment	This scale can be divided into seven subtests, including visuospatial/executive, naming, attention, language, abstraction, delayed recall, and orientation. The MoCA total score reflects the global cognitive performance.	Patients with MDD, patients with mild cognitive impairment	Unspecified, but it requires less time for completion than a complete set of neurocognitive tests
Harvey et al. (23)	UPSA	University of California San Diego performance-based skills assessment	It measures the everyday functioning skills in five function domains: comprehension/planning, finance, transportation, household, communication	Patients with MDD, patients with mild cognitive impairment	Unspecified
Ott et al. (29)	SCIP	Screen for cognitive impairment in psychiatry	SCIP consists of five subtests: verbal learning, working memory, verbal fluency, delayed memory, processing speed	Healthy controls, patients with bipolar disorder, MDD or schizophrenia	< 20 min
Ott et al. (29)	COBRA	The cognitive complaints in bipolar disorder rating assessment	16-item self-reported instrument, which allows measure subjective cognitive dysfunctions including executive function, processing speed, working memory, verbal learning and memory, attention/concentration and mental tracking. The COBRA total score is obtained when the scores of each item are added up.	Patients with bipolar disorder, unipolar depression	Unspecified
McIntyre and Lee (24)	THINC-it Tool		It includes the 5-item Perceived Deficits Questionnaire (PDQ-5) and four traditional cognitive assessments.	Patients with MDD	10–15 min
IsHak et al. (30)	RI	Recovery index	It is based on a combination of the WSAS and Q-LES-Q scales, provides information on the level of social, personal, and work functioning. It can be calculated through accessing a web platform	Non-specific	Unspecified

## Discussion

Although the paradigm of early diagnosis and individualized treatment represents the mainstay of the optimal management of patients with MDD (12), several unmet needs still exist and are reported by patients. In particular, cognitive dysfunctions represent a key determinant of functional disability in MDD patients (8, 31, 32) which can persist beyond clinical symptom remission (32), limiting work functioning, and contributing to the overall disability associated with MDD (22, 24, 33–38). It has been extensively reported that not paying attention to the cognitive dimension in patients with MDD may hamper the achievement of full recovery. For many years, cognition has been mainly evaluated in patients with other severe mental disorders, such as schizophrenia or bipolar disorders, and has not been considered a core dimension of the clinical presentation of patients with MDD. Nowadays, the establishment of the full functional recovery as new endpoint in the treatment of patients with MDD has highlighted the need to assess adequately cognitive symptoms and then, treat them.

The main finding of this clinical review is that several assessment tools exist for evaluating functional capacity. In particular, the UPSA has been useful to evaluate the everyday living skills, which is often a neglected aspect of other assessment tools (23). The UPSA gave the opportunity to evaluate the functional capacity, independently from mood symptoms. However, the UPSA has been developed in a pre-digital era and therefore its use may be overcome by modern technology. Therefore, other assessment instruments have been recently developed by including digital skills in the use of smartphones or devices, whose use has become widespread.

As regards the assessment of cognitive functioning, SCIP-D and COBRA are two new assessment tools recently validated in patients with MDD. In particular, the SCIP-D is very short and easy to use and therefore may be routinely administered in clinical practice; however, this instrument does not provide a full examination of neurocognitive functioning and it is better considered as a screening tool (39). The COBRA has a lower level of sensitivity and specificity compared to the SCIP-D for assessing objective cognitive dysfunctions; some authors have used a combined version of the two scales improving their validity (29).

The THINC-it TOOL (26) is a free-of-charge, digitalized, downloadable, application available for tablets and smartphones, which can be used in several clinical settings. Moreover, it is user-friendly and can be self-administered so that patients can regularly check their improvement in cognitive functioning. However, the need to be skilled in the use of smartphones or PCs may be a limitation, particularly in older patients. The MoCA is a reliable and valid instrument for measuring cognitive impairment in MDD patients. This instrument, which has already been translated in several languages, is quite short and requires lower time for completion compared to a complete set of neurocognitive tests. Both the MoCA and the SCIP-D (29) can be considered good screening tools for the evaluation of cognitive functioning.

The availability of instruments for the assessment of all the dimensions of cognitive functioning is probably a first step toward the shift in clinical practice from symptom remission to full functional recovery. In order to increase feasibility in routine care, these assessment tools should be easy to use and not time-consuming, as is the case with the Think-it tool or the SCIP-D. Also the “Recovery Index” may be implemented in routine care for the evaluation of psychosocial functioning of patients with MDD given its usefulness and easiness to use.

By assessing the cognitive functioning of patients with MDD, the positive impact of some pharmacological agents on these domains becomes clear. In the vast majority of patients, the treatment of cognitive symptoms represents a relevant problem in clinical practice, which impacts on the level of personal and cognitive functioning of patients. The use of tools focused on cognitive functioning or of tools with a mixed focus on social and cognitive functioning, such as the “Recovery Index,” should be promoted in clinical practice, considering the central role of cognitive functioning on the global level of functioning of patients with MDD.

Some conventional antidepressants mitigate cognitive symptoms in people with depression, but a significant proportion of antidepressants inhibit cognitive functioning (40, 41). Recently, the CANMAT guidelines (2016) (42) suggested to tailor the pharmacological treatment on the basis of clinical specifiers. In particular, for patients with cognitive dysfunctions, the following pharmacological compounds should be preferred: Vortioxetine (Level 1), Bupropion (Level 2), Duloxetine (Level 2), SSRIs (Level 2). According to the CANMAT, only vortioxetine, an antidepressant agent (43, 44) with a multimodal action mediated by the combination of a direct effect on serotonin receptor activity and reuptake inhibition of SERT (45, 46), has level 1 of evidence compared to other antidepressants for managing cognitive dysfunction (47, 48). Compared to other antidepressant agents, patients treated with vortioxetine report better cognitive functioning (49), and this improvement is independent from the improvement of affective symptoms (13).

Drugs targeting multiple neurochemical systems simultaneously (e.g., serotonin–noradrenaline reuptake inhibitors) might be more likely to improve cognitive performance than treatments targeting a single system only (e.g., selective serotonin reuptake inhibitors) (50, 51). In particular, bupropion has been tested in improving memory and mental processing speed performance (52). Duloxetine has been proven to be effective in improving cognitive score as compared to placebo, and this change was found to be independent from the ameloriation of affective symptoms (53).

On the other hand, the antagonism on M1, H1, and α1 receptors (as observed in the case of TCAs) have been hypothesized as impacting negatively on cognitive functioning (54–56).

Although the pharmacological treatment is essential for the succesfull management of patients with MDD, the complete recovery is not guaranteed, as shown by the occurrence of relapses and recurrences (57). For this reason, psychosocial interventions, such as psychoeducation, cognitive remediation, and cognitive-behavioral therapies, have been increasingly recognized as an essential component in the treatment of MDD, in association with pharmacological strategies (where needed), to achieve full recovery. In particular, it is necessary to integrate psychosocial treatment with pharmacological therapy, since these interventions are effective in improving the clinical course, treatment adherence, and psychosocial functioning of patients with MDD.

First-line psychological treatment recommendations for acute MDD include cognitive-behavioral therapy (CBT), interpersonal therapy (IPT), and behavioral activation (BA) (58). Whenever feasible, the combination of psychological interventions (CBT or IPT) with antidepressant treatment is recommended because combined treatment is superior to either treatment alone (58). First-line psychological treatments for maintenance phase include CBT and mindfulness-based cognitive therapy (MBCT). In order to select the type of psychosocial interventions, patient's preference for developing a personalized treatment plan shall be considered. A recent meta-analysis found that CBT is effective in patients with MDD, regardless of the baseline severity of the depressive episode, and can contribute to the achievement of full functional recovery (59). Several international guidelines suggest providing psychoeducational interventions to patients with MDD (60, 61). Different types of psychoeducational interventions are currently available, with the single-family approach, in which sessions are conducted with one family only, showing the most promising results (62). In particular, a systematic review has indicated that providing information about depression and its treatment is associated with a better prognosis and a reduction of family burden (63). Another psychosocial approach useful for achieving full functional recovery in patients with MDD is cognitive remediation (64–67). In particular, cognitive remediation—through the repeated activation of brain regions—can promote neuroplasticity, restoration of compromised neural processes, and improvement in neural function (68). Cognitive remediation programs include the repeated completion of cognitive tasks during several weeks. A recent meta-analysis (69) has confirmed that patients receiving this therapy report an improvement in attention, working memory and in the overall level of personal functioning. Furthermore, cognitive remediation seems to be the most promising intervention not only in improving cognitive functions, but also in improving depressive symptoms, contributing to the global recovery of the patients and to the full functional recovery (67). It is still debated the role of exercise interventions in improving cognitive functioning in patients with MDD (70). A recent meta-analysis (71) emphasized a lack of positive effect of physical exercise on cognition in patients with MDD. However, authors underlined that several limitations can have influenced their results, such as the small sample sizes of the included studies, the low dosage of physical exercises or the lack of cognitive assessment at baseline. Further studies are still needed in order to investigate the efficacy of psychosocial interventions, including physical activity component on cognitive functioning and full functional recovery of patients with MDD.

Other non-pharmacological strategies for improving cognitive symptoms in patients with MDD are repeated transcranial magnetic stimulation (rTMS) and transcranial direct current stimulation (tDCS) (72, 73). However, further studies are needed for evaluating the long-term efficacy of these treatments.

The present clinical review has some limitations which should be acknowledged. Given the nature of the included studies, we could not perform a meta-analysis, which would be however out of the scope of this paper. Moreover, this is not a systematic review, but it is rather a clinical review on recently developed assessment tools for evaluating psychosocial and cognitive functioning in patients with MDD. Another limitation is the short time frame for the inclusion of assessment tools. However, this methodological choice was made given the recent social and digital changes occurred in modern society. Finally, we did not search for gray literature, but we have focused on validated assessment tools only.

## Conclusions

Several assessment tools are available for evaluating the cognitive functioning in patients with MDD. Nevertheless, there is the need to promote further studies adopting homogenous assessment instruments, in order to explore the objective and subjective cognitive functioning.

Longitudinal studies with representative sample and control groups are needed for assessing the effects of antidepressant therapy and compare groups of different ages and evaluating the impact of gender differences on cognitive function. Regarding the cognitive remediation approach, more longitudinal studies on a wider variety of treatments are needed. Since psychotherapeuthic approaches have been found to be effective in improving cognition, when associated with antidepressant drugs, it should be useful to clarify the specific role of each treatment in obtaining this improvement.

Another relevant aspect is that the same treatment will not work for all patients with MDD (74) and when defining the treatment programme of MDD depression, clinicians should consider to tailor it to patients' needs and preference and to adopt a shared-decision making style, which has been proven to be effective in improving long-term outcomes (75–81). Moreover, as recently pointed out in a survey involving all the categories of stakeholders of mental health, there is the need to include users' perspective in research studies (82–87), and people with MDD have their preferences on treatment choice and want to be actively involved in discussion about their care.

Finally, the most relevant clinical implication of assessing social and cognitive functioning in routine care may be the real shift in the management of patients with MDD from symptom remission to full functioning recovery.

## Author contributions

The idea of the manuscript was conceived during a Lundbeck Advisory Board in 2017, attended by AF, BC, SDG, SLP, GM, ES, AF and AV; with the exception of GS. AF wrote the first version of the manuscript; GS, BC, SDG, SLP, GM, ES, AT, AV and AF revised all manuscript drafts having full control over content.

### Conflict of interest statement

The authors declare that the research was conducted in the absence of any commercial or financial relationships that could be construed as a potential conflict of interest. The reviewer NY and handling Editor declared their shared affiliation.

## Abbreviations

COBRA: cognitive complaints in bipolar disorder rating assessment
MoCA: montreal cognitive assessment
MDD: major depressive disorder
Q-LES-Q: quality of life enjoyment and satisfaction questionnaire
RI: recovery index
SCIP-D: screen for cognitive impairment in psychiatry-depression
UPSA: university of california san diego performance-based skills assessment
WSAS: work and social adjustment scale.
